# Detailed Structural Features of the Perovskite-Related Halide RbPbI_3_ for Solar Cell Applications

**DOI:** 10.1021/acs.inorgchem.1c03841

**Published:** 2022-03-28

**Authors:** Carmen Abia, Carlos A. López, Javier Gainza, João Elias F. S. Rodrigues, Mateus M. Ferrer, Gustavo Dalenogare, Norbert M. Nemes, Oscar J. Dura, José L. Martínez, María T. Fernández-Díaz, Consuelo Álvarez-Galván, José A. Alonso

**Affiliations:** † Instituto de Ciencia de Materiales de Madrid, CSIC, Cantoblanco, Madrid 28049, Spain; ‡ Institut Laue Langevin, BP 156X, Grenoble F-38042, France; § Instituto de Investigaciones en Tecnología Química (UNSL-CONICET) and Facultad de Química, Bioquímica y Farmacia, Almirante Brown 1455, San Luis 5700, Argentina; ∥ European Synchrotron Radiation Facility (ESRF), 71 Avenue des Martyrs, Grenoble 38000, France; ⊥ CCAF, PPGCEM/CDTec, Federal University of Pelotas, Pelotas 96010-610, Rio Grande do Sul, Brazil; # Departamento de Física de Materiales, Universidad Complutense de Madrid, Madrid E-28040, Spain; ¶ Departamento de Física Aplicada, Universidad de Castilla-La Mancha, Ciudad Real E-13071, Spain; ∇ Instituto de Catálisis y Petroleoquímica, CSIC, Cantoblanco, Madrid 28049, Spain

## Abstract

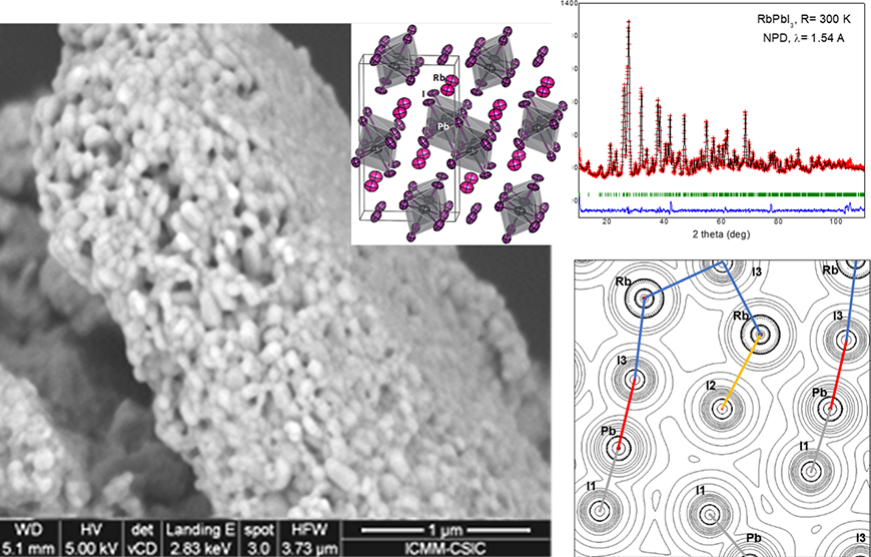

All-inorganic lead halide perovskites like CsPbBr_3_, CsPbI_3_, or RbPbI_3_ are good replacements for the classical hybrid organic–inorganic perovskites like CH_3_NH_3_PbI_3_, susceptible to fast degradation in the presence of humid air. They also exhibit outstanding light absorption properties suitable for solar energy applications. Here, we describe the synthesis of RbPbI_3_ by mechanochemical procedures with green credentials, avoiding toxic or expensive organic solvents; this specimen exhibits excellent crystallinity. We report neutron powder diffraction data, essential to revisit some subtle structural features around room temperature (200–400 K). In all these regimes, the orthorhombic *Pnma* crystal structure is characterized by the presence along the *b* direction of the crystal of double rows of edge-sharing PbI_6_ octahedra. The lone electron pairs of Pb^2+^ ions have a strong stereochemical effect on the PbI_6_ octahedral distortion. The relative covalency of Rb–I versus Pb–I bonds shows that the Pb–I-related motions are more rigid than Rb–I-related vibrations, as seen in the Debye temperatures from the evolution of the anisotropic displacements. The optical gap, measured by diffuse reflectance UV–vis spectroscopy, is ∼2.51 eV and agrees well with *abinitio* calculations. The thermoelectric Seebeck coefficient is 3 orders of magnitude larger than that of other halide perovskites, with a value of ∼117,000 μV·K^–1^ at 460 K.

## Abstract 2

All-inorganic RbPbI_3_ perovskites have been grown from a mechanochemical method under mild milling conditions. They yield a better-crystallized sample with appealing thermoelectric properties and a suitable band gap for solar cell applications.

## 1 Introduction

Perovskite solar cells (PSCs) are extremely appealing technologies for providing inexpensive solar electricity.^1−3^ For visible light conversion in photoelectrochemical cells, TiO_2_ can be efficiently sensitized by hybrid organic–inorganic halide perovskite nanocrystals, typically CH_3_NH_3_PbI_3_ (CH_3_NH_3_^+^: methyl ammonium cation, MA). Strong semiconducting band gap absorption is observed in nanocrystalline perovskites self-assembled on mesoporous TiO_2_ films. Significant power conversion efficiencies are measured under full sunlight in single-junction devices based on highly crystalline perovskite absorbers due to their intense visible to near-infrared absorptivity. In fact, the electron–hole diffusion length in a MAPbI_3_ absorber is ∼100 nm,^3^ enabling highly efficient planar heterojunction PSCs. Among other advantages, flat PSCs present an inherent suitability for flexible substrates, besides their ability to form hybrid silicon/perovskite tandems. Unfortunately, the paradigmatic MAPbI_3_ is unstable in humid air. An alternative is to combine it in a bilayer solar-cell architecture with either the narrower band gap, but also unstable, formamidinium lead iodide (FAPbI_3_) or with the more stable methylammonium lead bromide (MAPbBr_3_).^4^ Formamidinium-based perovskites also suffer from problems like the “yellow-phase impurity” (∂-FAPbI_3_), which can be suppressed via the addition of RbI, which in turn forms RbPbI_3_ impurities that turn out not to have detrimental effects and contribute to a higher mobility of the charge carriers with a longer useful lifetime, culminating in excellent 20.3 % power output.^5^

All-inorganic perovskites can provide an alternative to hybrid organic–inorganic perovskites, but they are not without hindrances. The compound with the most suitable band gap, the cubic phase of bulk CsPbI_3_ (α-CsPbI_3_), can only be stabilized at high temperatures. However, it can form nanoscale quantum dots (QDs) that work well in efficient optoelectronic devices.^6^ Crucially, α-CsPbI_3_ QD films do not deteriorate in ambient air. Colloidal perovskite QD photovoltaic cells form light-emitting diodes with an open-circuit voltage of 1.23 V. Together with other all-inorganic perovskite lead halides (CsPbX_3_, X = Cl, Br, I), the entire visible spectral region (410–700 nm) is accessible with bright photoluminescence (PL),^7^ and for photodetector devices that can be tuned in nanocrystals (NCs), by simple halide ion exchange reactions.^8^ Furthermore, it has been recently demonstrated that the presence of methylammonium in hybrid perovskites adds a significant source of nonradiative loss,^9^ stemming from the removal of a hydrogen atom from the organic molecule, which can be triggered by photons incident on the cell. This effect, really detrimental for efficiency, is not present in all-inorganic perovskites.

Promisingly, band gaps from first-principles calculations of lead halide perovskite semiconductors, including CsPbX_3_ (X = Cl, Br, I) and RbPbI_3_, agree very well with experimental values.^10−13^ The band gap of CsPbI_3_ makes it ideally suited for use in tandem solar cells.^14,15^ However, the ambient instability of CsPbI_3_ hampers its application in thin film form. For this reason, the much enhanced phase stability in environmental conditions of the Rb counterpart makes it a good light absorber candidate for use in solar cells and photodetectors, justifying further attention. Both CsPbI_3_ and RbPbI_3_ compounds share common features, such as the orthorhombic *Pnma* symmetry of the crystal structure and isotropic thermal expansion with nearly identical relative change of the lattice parameters.^16^ Yet, their structural evolutions differ strikingly near 600 K: at 602 K, CsPbI_3_ has a first-order reversible phase transformation *Pnma* → *Pm3̅m*, although RbPbI_3_ maintains its *Pnma* structure until it melts.^15^ PL studies show a PL center value of 2.1 eV at 300 K.^18,19^ The RbPbI_3_ perovskite-related halide has even been introduced into solar cells, taking advantage of the superior stability in environmental conditions, including a TiO_2_/RbPbI_3_ configuration.^17^ Even though the device performance with RbPbI_3_ is inferior compared to that with the cesium counterpart (open-circuit voltage of 0.62 V, photocurrent density of 3.75 mA·cm^–2^, and fill factor of 44.60%), this approach establishes the realization of highly stable perovskite films, achieving an incipient photovoltaic performance for real applications.

Recently, all-inorganic perovskite-type halides were synthesized by all-solid-state mechanochemical synthesis with various dimensionalities, as defined by the PbX_*n*_ polyhedra in three (3D), two (2D), and zero (0D) dimensions: 3D CsPbBr_3_, 2D CsPb_2_Br_5_, 0D Cs_4_PbBr_6_, 3D CsPbCl_3_, 2D CsPb_2_Cl_5_, 0D Cs_4_PbCl_6_, 3D CsPbI_3_, and 3D RbPbI_3_.^20^ In the latter case, nevertheless, the sample was not structurally characterized; moreover, the RbPbI_3_ halide exhibits great potential for quantum dots applications,^21^ making necessary a profound study on its crystal structure. Transport properties, such as the Seebeck coefficient and thermal conductivity, remain barely known for RbPbI_3_. As similar families of halides are being studied for possible thermoelectric applications,^22−25^ it is of great importance to shed light on the mentioned properties.

For this reason, here, we present temperature-dependent (200–400 K) neutron powder diffraction (NPD) data to study the structural evolution of highly crystalline RbPbI_3_ prepared by mechanochemistry. We confirm the existence of an orthorhombic *Pnma* phase that persists in the whole temperature range. The NPD data allow determining highly accurate values of the displacement factors. The analysis of these displacement factors unveils that Pb–I bonds are more rigid than the Rb–Cl bonds, while theoretical topochemical evaluations disclose a relevant covalent contribution for the Pb–I pair bond. Furthermore, scanning electron microscopy (SEM), differential scanning calorimetry (DSC), optical spectroscopy, and thermoelectric and thermal conductivity characterization complement the structural study. The Seebeck coefficient above room temperature (RT) is exceptionally high, around 44,000 μV·K^–1^ at 400 K.

## 2 Experimental Methods

RbPbI_3_ was obtained as a microcrystalline powder from mechanosynthesis in a planetary ball mill, from stoichiometric amounts of RbI (Strem) and PbI_2_ (Alfa-Aesar), working in N_2_ atmosphere. 1.5 g of the reactants was milled using 30 zirconia balls of 5 mm diameter, with a final 8.6:1 mass ratio, for 4 h at 450 rpm in a Retsch PM100 mill. A laboratory X-ray diffraction (XRD) pattern was collected on a Bruker D5 diffractometer with Cu K_α_ (λ = 1.5418 Å) radiation. In order to investigate the crystallographic structure, a NPD study was carried out at 200, 250, 300, 350, and 400 K in the D20 instrument (Institute Laue Langevin, Grenoble, France) with a wavelength of 1.540 Å. The sample, contained in a V cylinder, was introduced in a cryo-furnace; the patterns were collected for 30 min each. The refinement of the crystal structure was performed by the Rietveld method using the *Fullprof* software.^26,27^ A pseudo-Voigt function was chosen to generate the line shape of the diffraction peaks. The background was interpolated between regions devoid of reflections. The following parameters were refined in the final run: scale factor, background coefficients, zero-point error, pseudo-Voigt corrected for asymmetry parameters, positional coordinates, anisotropic displacement factors, and occupancy factors. For the neutron refinements, the coherent scattering lengths for Rb, Pb, and I were 7.090, 9.405, and 5.280 fm, respectively; these distinct values guarantee a precise determination of the atomic positions. Moreover, as the scattering length values do not decay with the diffraction angle, intense peaks are obtained at high angles, thus improving the accuracy of the displacement factors. DSC measurements were carried out in the range 100–300 K with a heat pulse method. Field-effect SEM (FE-SEM) images were obtained in a FEI-Nova microscope, with an acceleration potential of 5 kV, coupled to an energy-dispersive X-ray spectrometry (EDXS) device, working with an acceleration voltage of 18 kV and 60 s of acquisition time. The optical diffuse reflectance spectrum of the RbPbI_3_ powder was measured at RT using a UV–vis spectrophotometer Varian Cary 5000.

In order to measure the transport properties, the powder was pressed to a pellet shape with no applied heat, using a cold press. The thermoelectric properties were measured in the resulting pellet, with neither sintering/annealing nor any other step in between. Seebeck coefficient was obtained by measuring simultaneously the drop voltages across the sample and a constant reference wire with an electrometer (Keithley 6517B) and a nanovoltmeter (Keithley 2182A) under vacuum (10^–3^ mbar). The electrical resistivity was measured using an Agilent E4980A LCR meter. The total thermal conductivity was calculated from the thermal diffusivity (α) using a Linseis LFA 1000 equipment, by the laser-flash technique. The thermal conductivity (κ) was determined using κ = α × *c*_p_ × *d*, where *c*_p_ is the specific heat and *d* is the sample density.

## 3 Computational Methods

The quantum models were elaborated according to density functional theory (DFT) with PBE functional^28^ using *CRYSTAL17* package.^29^ The basis set of rubidium (Rb), lead (Pb), and iodine (I) was defined using the *POB*-*TZVP* basis developed by Laun and co-workers.^30^ The Coulomb and exchange series thresholds (overlap and penetration for Coulomb integrals, the overlap for HF exchange integrals, and the pseudo-overlap) of the package were set as 10^–8^, 10^–8^, 10^–8^, 10^–8^, and 10^–16^, respectively. The shirking factors (Pack–Monkhorst and Gilat net) were set as 6 and 6, respectively. In the structure optimization step, the gradient components and nuclear displacements were adjusted with tolerances on their root-mean-square of 0.0003 and 0.0012 a.u., respectively. The main bond critical points (BCPs) of the structures were evaluated, according to “quantum theory: atoms in molecules” (QTAIM), in order to assist in understanding the electronic characteristics of the chemical bonds. The QTAIM was carried out with the *TOPOND* program within the *CRYSTAL17* package.^31^ The crystal representation was carried out by *VESTA* software.^32^

## 4 Results and Discussion

### 4.1 Initial Characterization

**Figure 1:**
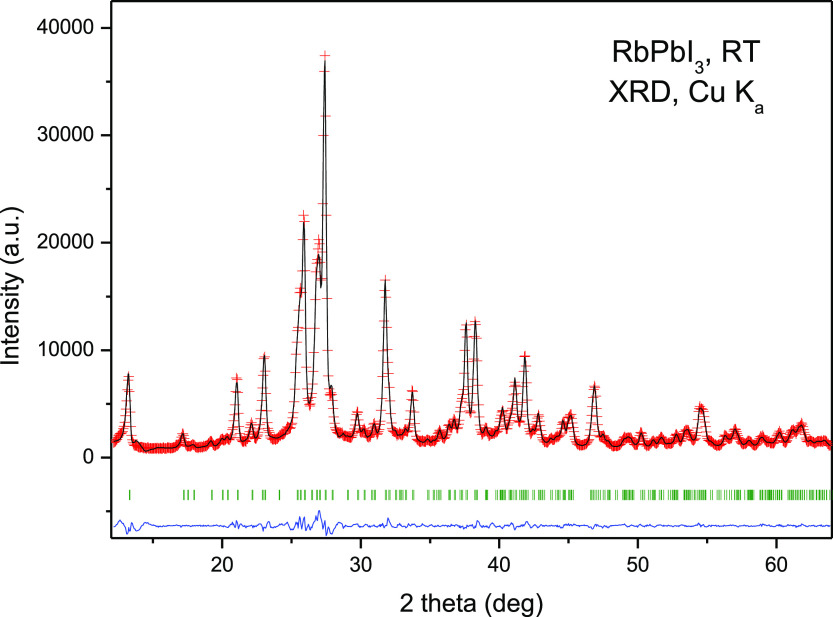
RT Rietveld plot from the laboratory XRD patterns of RbPbI_3_, prepared by ball milling.

RbPbI_3_ was obtained as a yellowish polycrystalline powder. Laboratory XRD was used for initial crystallographic identification at RT, with orthorhombic tetragonal symmetry, in agreement with previous reports, indexable in the space group *Pnma* (SG: #62). It belongs to the NH_4_CdCl_3_ structural type.^16^ The crystal structure was preliminarily refined in this structural model from laboratory XRD data, as displayed in Figure 1, obtaining as unit-cell parameters *a* = 10.2924(6) Å, *b* = 4.7795(2) Å, *c* = 17.4049(9) Å, and *V* = 856.19(8) Å^3^. These parameters are slightly larger than those reported, of *a* = 10.2761(9) Å, *b* = 4.7793(4) Å, *c* = 17.3933(12) Å, and *V* = 854.23 Å^3^.

**Figure 2:**
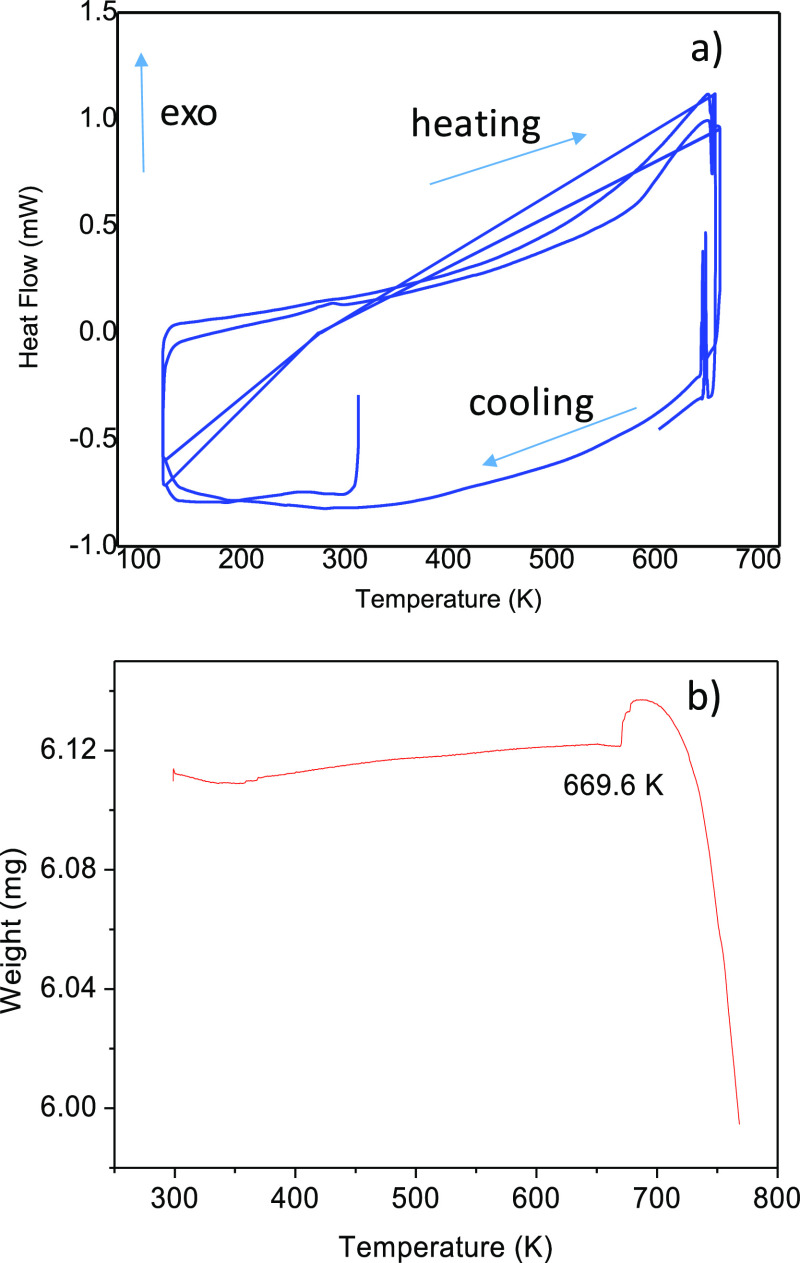
(a) Several cycles of DSC curves. The sharp, reversible peak observed at 654 K (heating) and 647 K (cooling) corresponds to the fusion of the sample. (b) TG curve, showing weight loss upon the decomposition of the sample above 670 K.

Figure 2a,b illustrates the DSC curves (in the heating and cooling runs) and the thermogravimetry (TG) curve. No significant events are identified in the calorimetric curve in the 130–640 K temperature range, except a sharp endothermic peak observed at 654 K (heating run) and the corresponding exothermic event at 647 K (cooling run), corresponding to the fusion/crystallization of the sample. In Figure 2b, the kink at 669.6 K also corresponds to the fusion process; the weight loss observed above 690 K indicates the full decomposition of the sample, by iodine loss.

### 4.2 Structural Characterization from NPD Data

**Figure 3:**
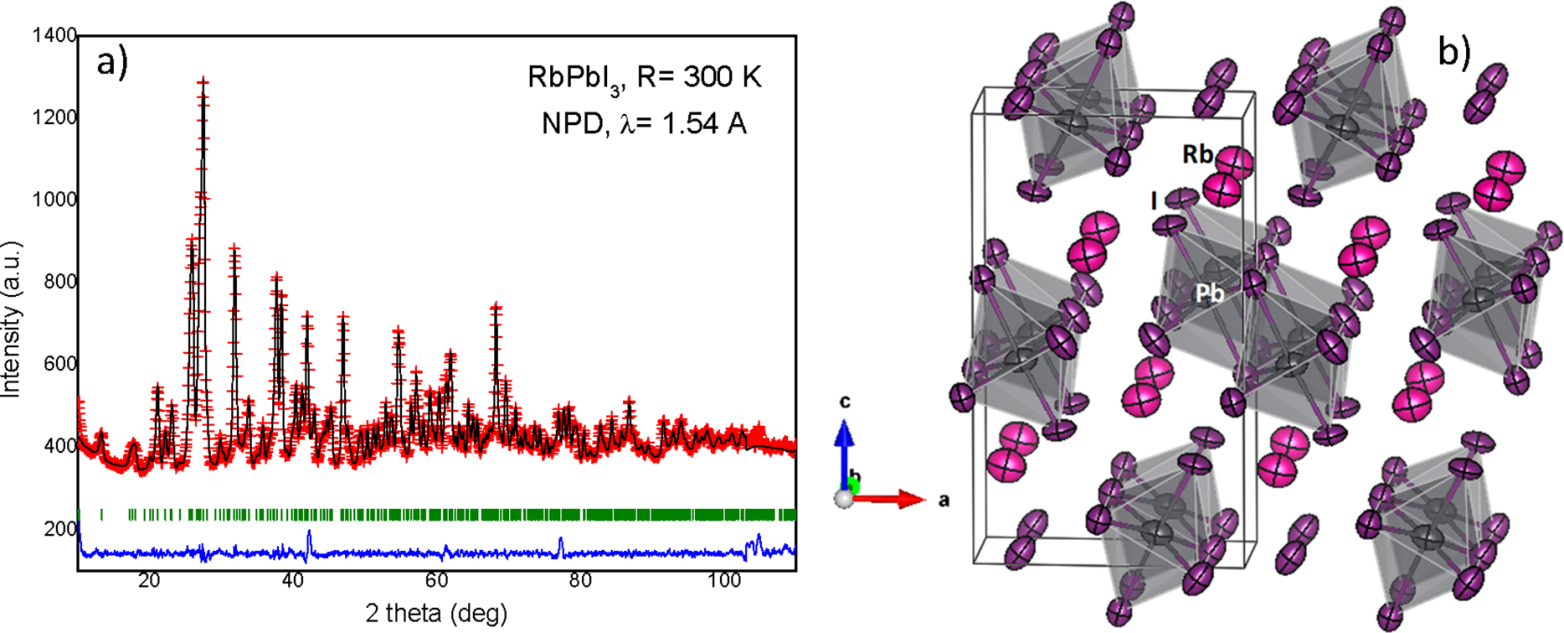
(a) Observed (crosses), calculated (black line), and difference (blue line) profiles after the Rietveld refinement in the *Pnma* structure from NPD data at 300 K. (b) View of the crystal structure enhancing the arrangement of PbI_6_ octahedra in double rows directed along the *b* direction and the anisotropic displacement factors.

The structural data published in the literature correspond to refinements from the XRD data [ref (16)], but there are no available measurements from NPD data. In order to perform a precise refinement from NPD data, the crystal structure was modeled in the mentioned *Pnma* space group. The Rb^+^, Pb^2+^ cations, and the three types of I^–^ anions (I1, I2, and I3) are all allocated at 4*c* (*x*, 1/4, *z*) Wyckoff sites. Figure 3 illustrates the quality of fit from NPD data at 300 K, including the refinement of the anisotropic displacement parameters for all the atoms. The remaining Rietveld plots are included in the Supporting Information. Table 1 lists the main crystallographic data. Figure 3 also includes a view of the crystal structure. The structure is three-dimensional, consisting of double rows of PbI_6_ octahedra sharing edges, directed along the *b* direction of the crystal, with the Rb^+^ ions in the interstices in between the octahedra, in ninefold coordination, with the Rb–I bond lengths spanning from 3.079(4) to 4.091(5) Å, at 300 K. Each PbI_6_ octahedron is composed of a Pb–I1 bond length (3.036(4) Å), two Pb–I2 distances (2 × 3.222(3) Å), and three Pb–I3 bond lengths (3.397(4) Å, 2 × 3.248(3) Å), at 300 K.

**Table 1:** Crystallographic Data for RbPbI_3_ Phase in the Orthorhombic System (*Pnma*) from NPD Data at 300 K^a^

	*x*	*y*	*z*	U_iso_^*^/U_eq_	Occ
Rb	0.4125(3)	0.25	0.6746(2)	0.053 (3)	1
Pb	0.1663(2)	0.25	0.4393(1)	0.0340 (16)	1
I1	0.30625(4)	0.25	0.2852(2)	0.039 (3)	1
I2	0.1599(4)	0.25	0.01023(2)	0.041 (3)	1
I3	0.0267(4)	0.25	0.6168(2)	0.031 (3)	1
Discr. factors: *R*_p_ = 0.91%, *R*_wp_ = 1.30%, *R*_exp_ = 0.73%, χ2 = 3.16, and *R*_Bragg_ = 1.89%					

atomic displacement parameters (Å^2^)						
	***U***^***11***^	***U***^***22***^	***U***^***33***^	***U***^***12***^	***U***^***13***^	***U***^***23***^
Rb	0.065 (2)	0.041 (3)	0.052 (3)	0.00000	0.003 (2)	0.065 (2)
Pb	0.0442 (17)	0.0317 (15)	0.0261 (15)	0.00000	0.0040 (16)	0.00000
I1	0.049 (3)	0.049 (3)	0.019 (3)	0.00000	0.007 (2)	0.00000
I2	0.040 (3)	0.030 (3)	0.051 (4)	0.00000	0.019 (3)	0.00000
I3	0.026 (3)	0.030 (3)	0.037 (3)	0.00000	–0.004 (2)	0.00000

a *a* = 10.2589(3) Å, *b* = 4.7679(1) Å, *c* = 17.3579(5) Å, and *V* = 849.03(4) Å^3^.

The octahedral distortion was calculated using the “distortion index” which is defined as
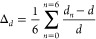
1where *d*_*n*_ and *<d>* are Pb–I_*n*_ and <Pb–I> distances, respectively.^33^ The value obtained in this case is Δ_*d*_ = 10.6 × 10^–4^, defining a considerable distortion that evidences the effect of the lone electron pair of Pb^2+^ ions.

### 4.3 200–400 K Neutron Diffraction Characterization

**Figure 4:**
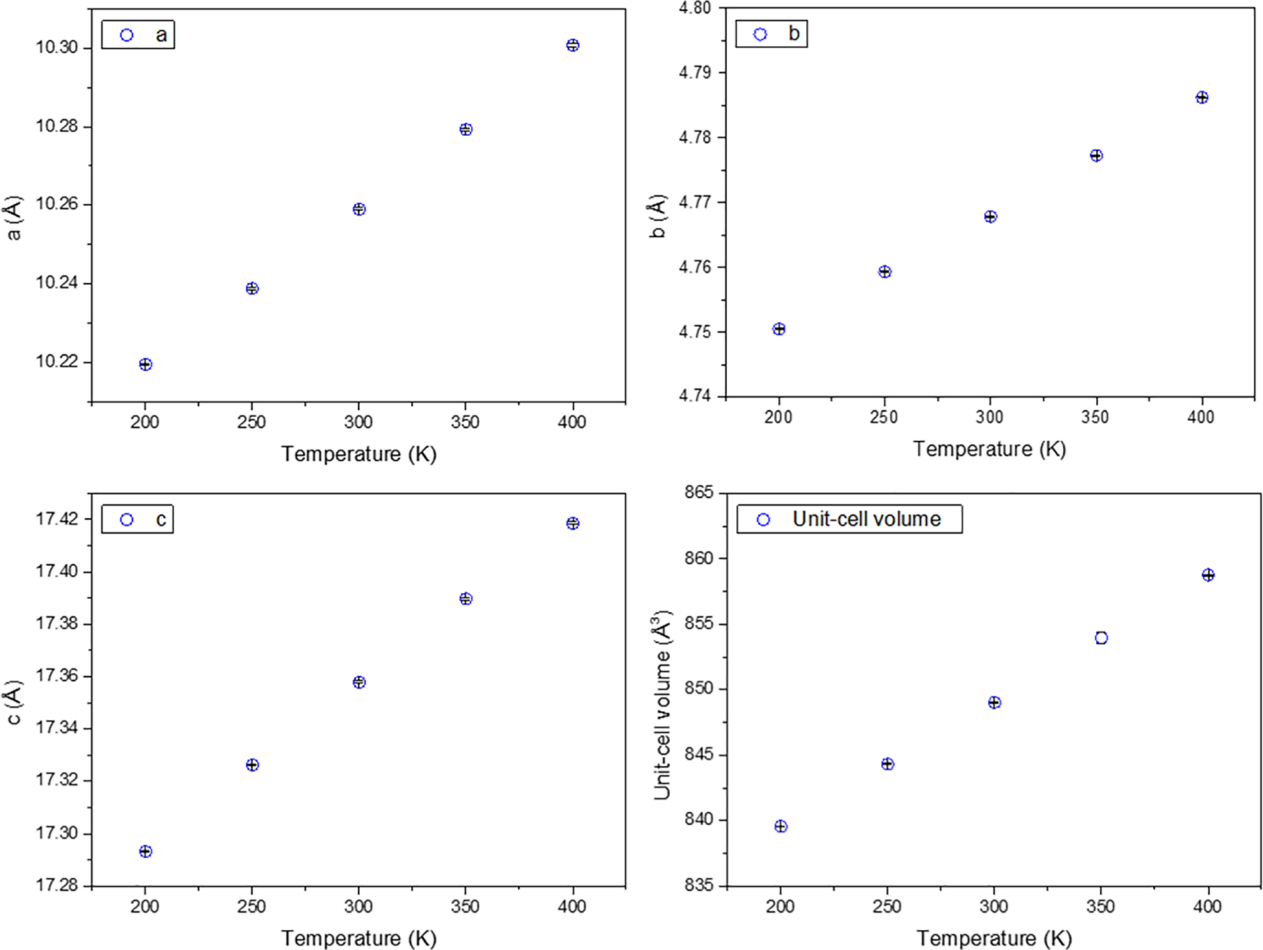
(a) *a*, (b) *b*, and (c) *c* unit cell parameters and (d) volume thermal evolution from temperature-dependent NPD data.

Temperature-dependent NPD patterns were measured at 200, 250, 350, and 400 K, showing that the orthorhombic unit cell is maintained in all the temperature ranges. Figure 4 shows the variation of *a*, *b*, *c*, and *V* unit cell parameters. All parameters regularly increase upon warming up, as expected from the thermal expansion. From the volume evolution, a thermal expansion coefficient of 39.1 × 10^–6^ is determined in the 200–400 K temperature range. The Rietveld plots and the crystallographic data at 200 and 400 K are displayed in Figures S1 and Tables S1 and S2 of the Supporting Information, respectively.

### 4.4 Mean-Square Displacements

The moderate absorption of neutrons by the heavy Rb and Pb atoms was suitable to probe the thermal variation of the mean-square displacement factors (MSDs) in the temperature range 200–400 K. Here, the MSDs were analyzed in their equivalent displacement parameters (*U*_eq_, in units of Å^2^), as derived from the anisotropic coefficients *U*^11^, *U*^22^, *U*^33^, *U*^12^, *U*^13^, and *U*^23^ for each atom within the *Pnma* crystal structure. The Debye model is supposed to describe the temperature evolution of the MSDs, as summarized below
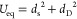
2where
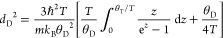
3such that *d*_s_^2^ is the intrinsic disorder, θ_D_ is the Debye temperature, *m* is the atom’s mass, and *T*, *k*_B_, and ℏ keep their usual meaning.^34,35^ As low-temperature points (<100 K) were not taken, the uncertainty associated with *d*_s_^2^ is high, when it is considered as a fitting parameter. In order to avoid this, such a parameter was kept equal to zero for all the atoms (Rb, Pb, I1, I2, and I3).

**Figure 5:**
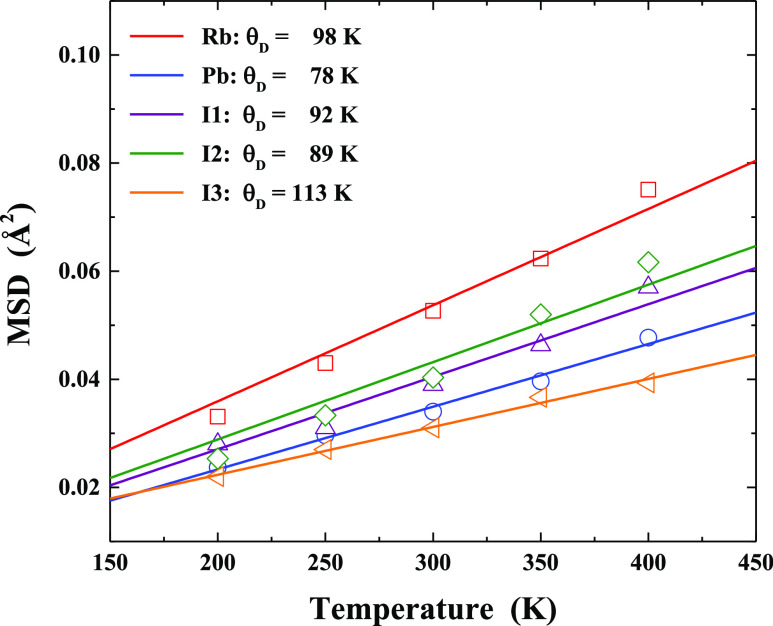
Temperature-dependent MSDs (open symbols) together with the fitting using the Debye model (lines).

Figure 5 exhibits the temperature dependence of MSDs for the atoms in the asymmetric unit within the RbPbI_3_ crystal structure. The Debye temperatures derived are listed in Table 2. The energy range of such values can be assigned to phonon modes, which typically have low values, that is, in the range 5–15 meV (∼40–120 cm^–1^), in halide perovskites.^36^ The bonding stiffness may be evaluated from the Debye temperature by considering the harmonic one-particle potential (OPP).^34^ Here, the harmonic potential is written as *V*_opp_(*d*_D_^2^) = 1/2*Kd*_D_^2^, where
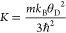
4

**Table 2:** Debye Temperature (θ_D_) and Force Constant (*K*) Estimated from the Harmonic OPP Model

atom	θ_D_ (K)	*k*_B_θ_D_ (meV)	*K* (eV·A^–2^)
Rb	97.7	8.4	0.48
Pb	77.7	6.7	0.74
I1	92.3	7.9	0.64
I2	89.4	7.7	0.60
I3	113.5	9.8	0.97

We have obtained the following values of *K* (see Table 2): 0.48 eV·Å^–2^ for Rb, 0.74 eV·Å^–2^ for Pb, 0.64 eV·Å^–2^ for I1, 0.60 eV·Å^–2^ for I2, and 0.97 eV·Å^–2^ for I3. Therefore, one may conclude that the Pb–I-related motions are more rigid than Rb–I-related vibrations, which is associated with the covalency exhibited between Pb and I atoms. From these low values of force constants and Debye temperatures, being of intrinsic origin, ultralow thermal conductivity might be expected for RbPbI_3_.

**Figure 6:**
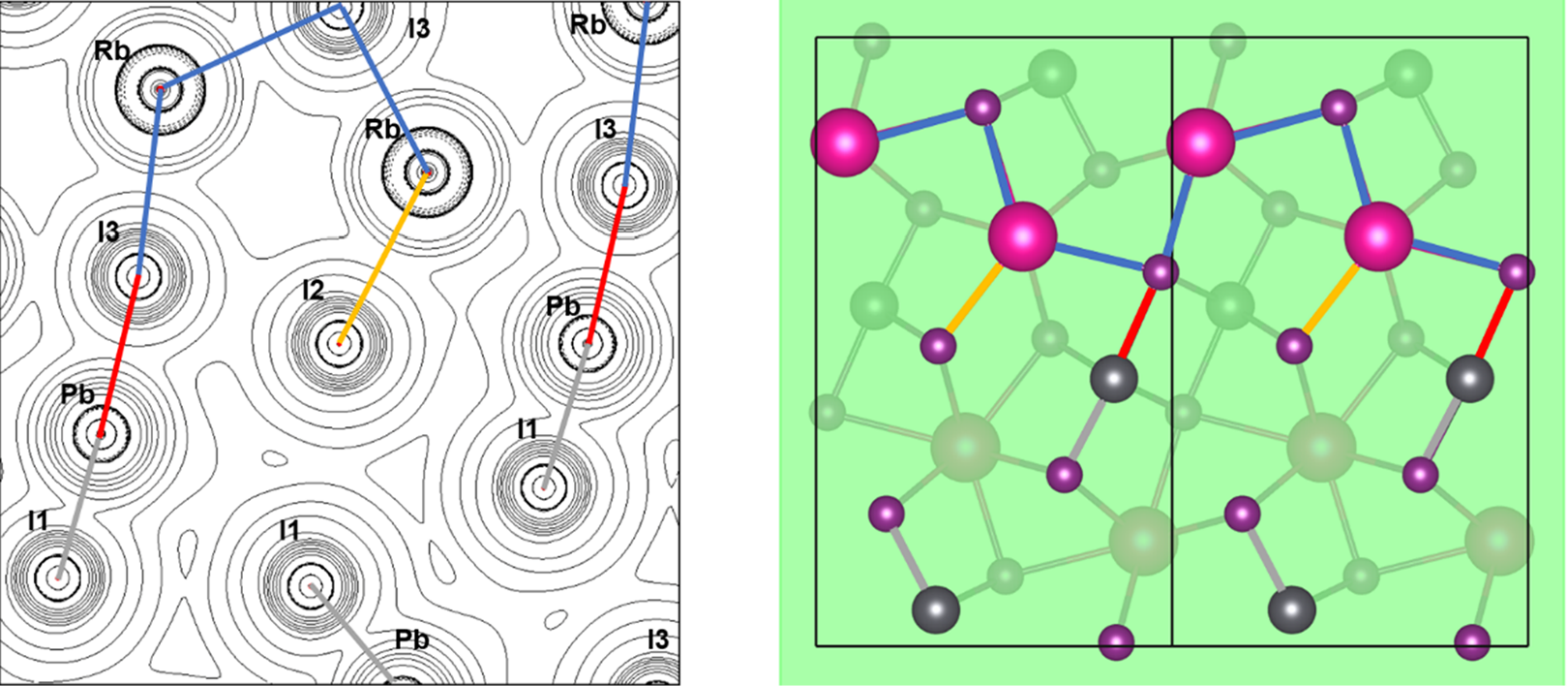
Laplacian of electronic density of plane (0 4 0) of the RbPbI_3_ model and the representative plane in the unit cell. Representative bonds of Rb–I2, Rb–I3, Pb–I1, and Pb–I3 in yellow, blue, gray, and red, respectively. The pair bonds Rb–I1 and Pb–I2 are out of the plane.

The topochemical analysis of the main bond critical points (BCPs) was performed to evaluate the chemical environment of RbPbI_3_. The estimated topochemical parameters are listed in Table 3. According to the data in the table, the low values of ρ and the positive values of ∇^2^ρ in all the BCPs indicate that all the bonds have a predominant ionic character. On the other hand, some behaviors are different for the BCPs between Rb–I and Pb–I bonds. The evaluation of the *H* parameter shows the positive values of the BCPs attributed to the Rb–I bonds and negative values of the ones attributed to the Pb–I bonds. In addition, the values of |*V*|/*G* for Pb–I bonds are greater than 1 (and less than 2), while the |*V*|/*G* values of the Rb–I bonds are less than 1. These different behaviors of the BCPs of the Pb–I bonds indicate that such bonds fall into a transient class showing a relevant covalent contribution,^37,38^ as estimated from the Debye analysis above. From a relative comparison, it is observed that the Rb–I bond has a greater ionic character than the Pb–I bond, results that corroborate the values indicated in the obtained results for the Debye temperature. The Laplacian of electronic density isolines with the atomic site’s information is depicted in Figure 6.

**Table 3:** Topochemical Parameters for RbPbI_3_ at Critical Points: Electron Density (**ρ)**, Laplacian of Electron Density (∇^2^**ρ)**, Virial Field Density (*V*), Lagrangian Kinetic Energy Density (*G*), and Total Energy (*H*)

pair bond	**Ρ**	∇^2^**ρ**	*G*	*V*	*H*	*|V|/G*
Rb–I1	7.33 × 10^–3^	2.23 × 10^–2^	4.60 × 10^–3^	–3.63 × 10^–3^	9.74 × 10^–4^	7.88 × 10^–1^
Rb–I2	5.37 × 10^–3^	1.72 × 10^–2^	3.38 × 10^–3^	–2.45 × 10^–3^	9.31 × 10^–4^	7.25 × 10^–1^
Rb–I3	6.06 × 10^–3^	1.97 × 10^–2^	3.93 × 10^–3^	–2.92 × 10^–3^	1.00 × 10^–3^	7.44 × 10^–1^
Pb–I1	2.42 × 10^–2^	3.80 × 10^–2^	1.14 × 10^–2^	–1.33 × 10^–2^	–1.87 × 10^–3^	1.16 × 10^0^
Pb–I2	3.62 × 10^–2^	5.06 × 10^–2^	1.75 × 10^–2^	–2.23 × 10^–2^	–4.84 × 10^–3^	1.28 × 10^0^
Pb–I3	2.90 × 10^–2^	4.24 × 10^–2^	1.36 × 10^–2^	–1.65 × 10^–2^	–2.94 × 10^–3^	1.22 × 10^0^

### 4.5 Thermoelectric Properties

**Figure 7:**
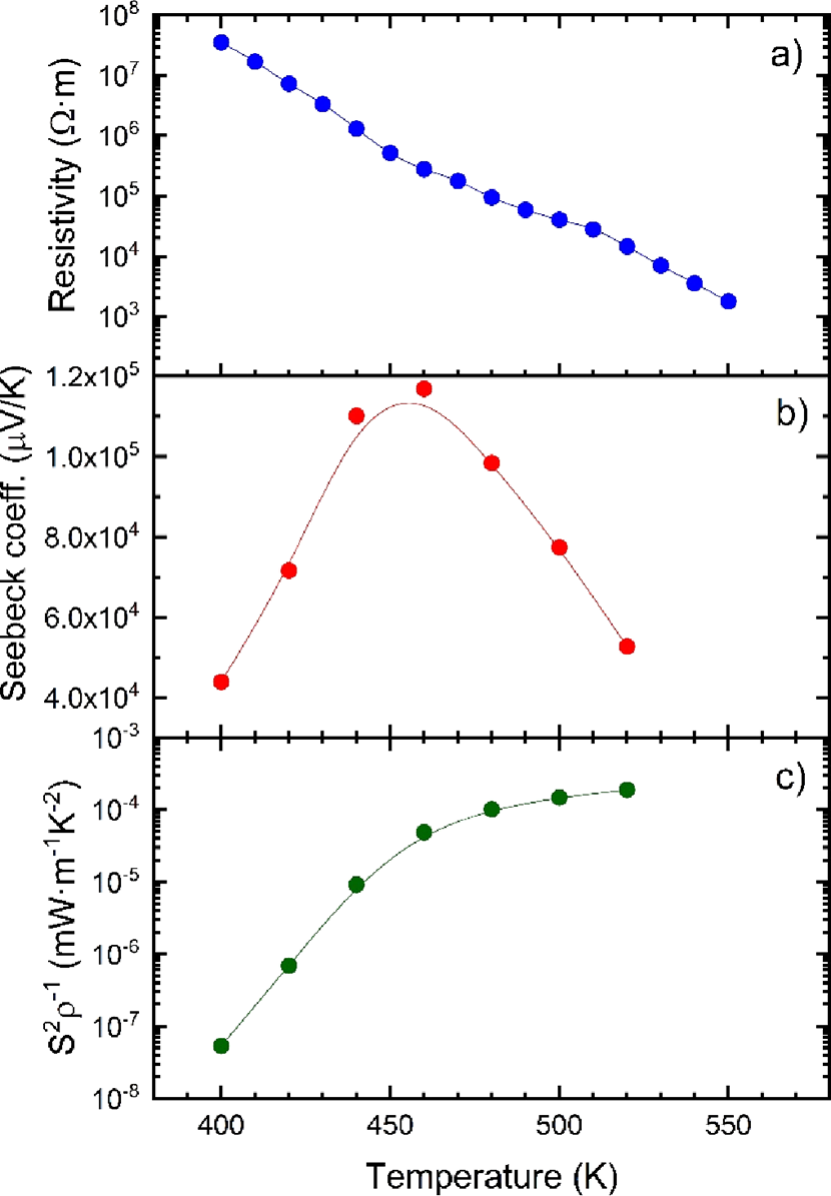
(a) Resistivity, (b) Seebeck coefficient, and (c) power factor of RbPbI_3_.

Figure 7 shows the three main thermoelectric quantities up to 550 K: resistivity (ρ = σ^–1^) (Figure 7a), Seebeck coefficient (*S*) (Figure 7b), and power factor (Figure 7c), defined as *S*^2^σ. The resistivity is remarkably high near RT, about 3 × 10^7^ Ω·m at 400 K, a really high value if we compare it to other halide perovskites.^22^ However, this magnitude is closer to the resistivity reported for single crystalline MAPbBr_3_,^25^ which shows a resistivity of about ∼3.3 × 10^6^ Ω·m. The resistivity decreases constantly with temperature, which is probably caused by the thermal activation of minority carriers, reaching 1.8 × 10^3^ Ω·m at 550 K.

The Seebeck coefficient strongly varies with temperature, showing remarkably high values, rarely seen in thermoelectric materials.^39^ At 400 K, the Seebeck coefficient is ∼44,000 μV·K^–1^. This value increases up to 117,000 μV·K^–1^ at 460 K, and immediately afterward, decreases again down to 53,000 μV·K^–1^ at 520 K. Even with this high Seebeck coefficient, which could predict a good thermoelectric performance, the resistivity is high enough to hamper the power factor, which shows values not higher than 2 × 10^–4^ mW·m^–1^·K^–2^. These types of materials can be appropriately doped to modify their carrier density,^22^ altering their resistivity and Seebeck coefficient conveniently.

**Figure 8:**
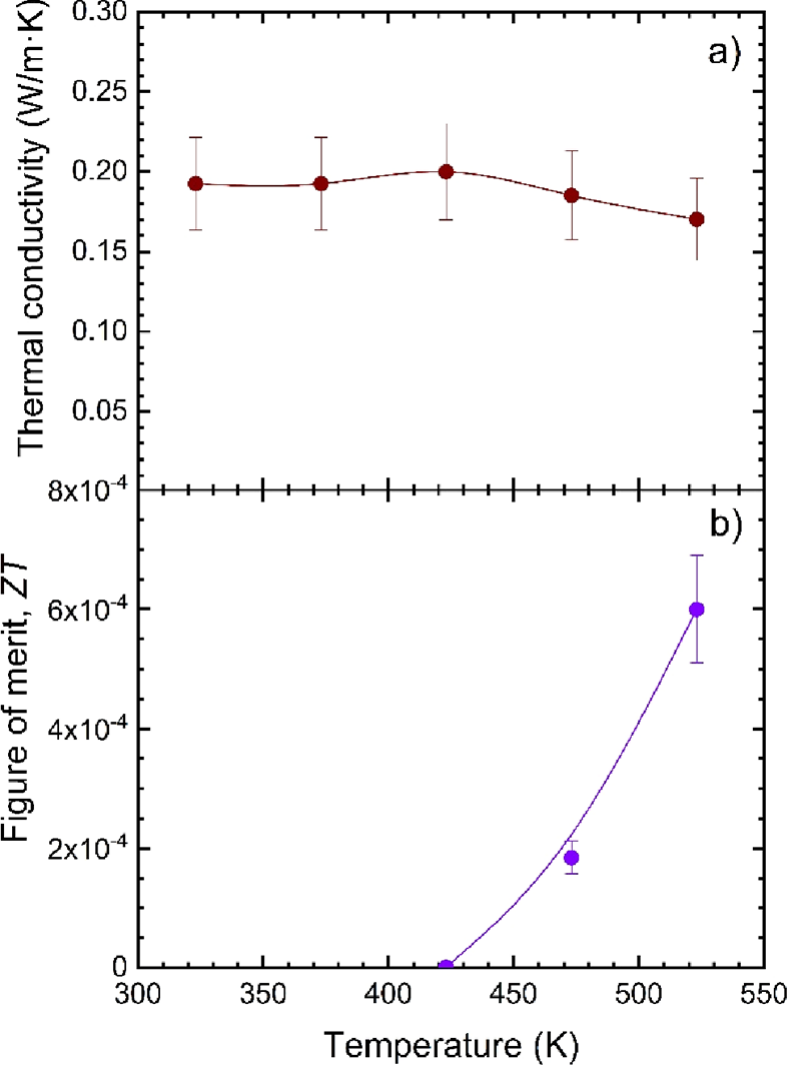
(a) Thermal conductivity and (b) thermoelectric figure of merit of RbPbI_3_.

On the other hand, the thermal conductivity κ (Figure 8a) is lower than that reported for other halide and hybrid perovskites,^22,25^ remaining always below 0.2 W·m^–1^·K^–1^ at all the measured temperatures, from 323 K up to 573 K. This nearly constant evolution with temperature has been seen before in hybrid organic–inorganic perovskite single crystals^25^ and in the RbPb_2_Br_5_ perovskite prepared by mechanochemistry.

The combination of these parameters in the thermoelectric figure of merit, *ZT* (*S*^2^σ*T*/κ), yields the result displayed in Figure 8b. This figure of merit is low compared to most thermoelectric materials, but at 523 K, it reaches 6 × 10^–4^, which is 2 orders of magnitude higher than the figure of merit reported for other halide perovskites, such as Bi-doped MAPbBr_3_,^25^ MASnBr_3_,^40^ and RbPb_2_Br_5_.

### 4.6 Microstructure by Scanning Microscopy (FE-SEM)

**Figure 9:**
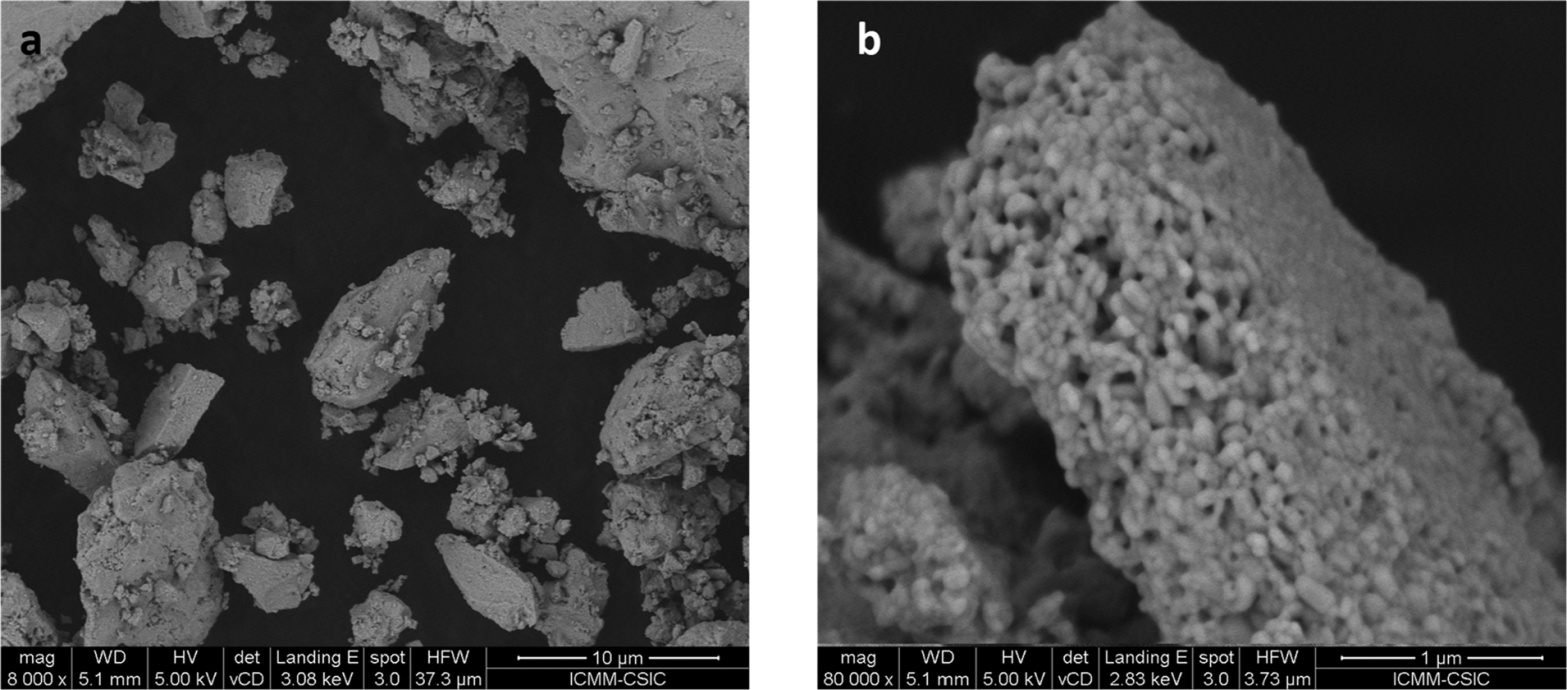
FE-SEM images of RbPbI_3_ samples at ×8,000 (a) and ×80,000 (b) magnifications.

In a mechanosynthesis process, as high-energy ZrO_2_ balls impact against the reactants, a highly disaggregated product of small particles is expected. However, SEM images of the as-prepared RbPbI_3_ polycrystals show a heterogeneous mixture of large agglomerates (10–20 μm) along with smaller fragments (Figure 9a). It can be related to the possible local cold sintering as a result of high-momentum transfer to the powder from the milling balls (typically, the local pressure could reach up to 6 GPa, with temperatures up to 473 K).^41^ Yet, at higher magnification (Figure 9b), much smaller grains are revealed to form the agglomerates, with typical sizes of 70–200 nm. Assuming monocrystalline individual grains, the diffraction volume is large enough to give the good crystallinity seen in XRD and neutron diffraction. Figure S2 shows the EDX spectrum with the atomic assignment, which reasonably coincides with the expected atomic ratio of the three elements (Rb, Pb, and I).

These powders resulting from ball milling can be dispersed in a particular medium (e.g., isopropyl alcohol) in order to be used for drop-coating-based thin films^42^ or spin-coating technique.^43^ As the stability of the powder sample is higher than that compared to the solvent-based method, as reported previously by our group,^44^ there is a good chance that the thin films could also show an enhanced stability.

### 4.7 UV–Vis–NIR Spectra

**Figure 10:**
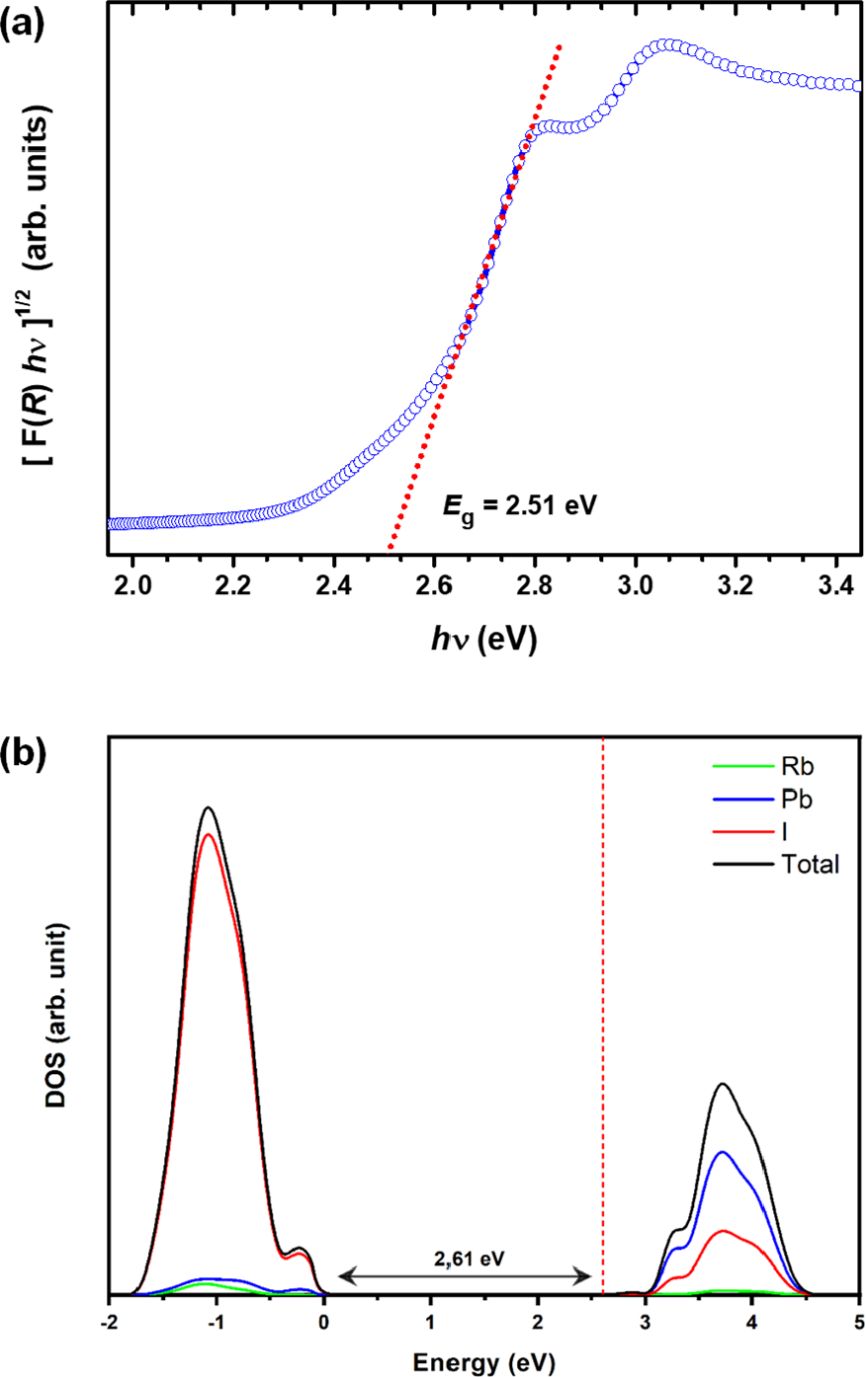
(a) Kubelka–Munk (KM)-transformed diffuse reflectance spectrum of RbPbI_3_. (b) DOS of the RbPbI_3_ model.

The optical absorption characteristics, in particular the band gap, of RbPbI_3_ powder were determined by diffuse reflectance UV–vis spectroscopy. The Kubelka–Munk function [*F*(*R*) = (1 – *R*)^2^/2*R*, where *R* is the measured reflectance] is closely related to the optical absorption coefficient (Figure 10). The energy gap, *E*_g_, was estimated by the Tauc method, as follows
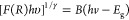
5where γ depends on the nature of electron transition, namely, 1/2 or 2 for direct or indirect transition energy gaps, respectively. For the reasons explained below, here, γ = 2 was chosen for an indirect transition, and *E*_g_ was estimated by linear extrapolation of the steepest edge. The value obtained for RbPbI_3_ (∼2.51 eV) is in close agreement with that obtained by *ab initio* calculations for the orthorhombic structure, *Pnma*, called δ-RbPbI_3_, of 2.663 eV.^11^ Using the previously described DFT model, we have also determined the density of states (DOS) with the contribution of each element of RbPbI_3_, as well as the electronic band gap, as depicted in Figure 10b.

As shown in Figure 10b, the valence band is composed mostly of iodine states, as expected for the most electronegative element. Furthermore, the conduction band has a higher contribution of the lead states, followed by a significant contribution of iodine states. This projection also shows a band gap value of 2.61 eV, close to the estimated band gap by UV–vis spectroscopy. In addition, the RbPbI_3_ model indicates an indirect band gap transition, as seen in the band structure (see in Figure S3 of Supporting Information).

## 5 Conclusions

A well-crystallized RbPbI_3_ material was obtained by mechanosynthesis using mild ball-milling. The unit cell parameters at RT are slightly larger than those described for samples synthesized by conventional solid-state reaction methods. The detailed crystal structure was refined from NPD in the 200–400 K temperature range. In this whole stability range, the crystal structure is orthorhombic, as defined in the *Pnma* space group; the lattice parameters and volume linearly increase with temperature. The bonding stiffness was estimated using the harmonic OPP from the Debye temperature analysis of the anisotropic MSD parameters, showing that the Pb–I bonds are more rigid than the Rb–Cl bonds. This result was corroborated by theoretical topochemical evaluations, in which the transient character of the Pb–I pair bonds was foreseen with a relevant covalent contribution. The thermoelectric properties are appealing, considering the large Seebeck coefficient and low thermal conductivity, and may expand the possibilities for future thermoelectric applications, although the large electrical resistivity limits the thermoelectric figure of merit. A band gap of ∼2.51 eV (indirect transition) is suitable for solar cell applications.

## Supporting Information Available

Rietveld plots from NPD data at 200 and 400 K; EDX spectrum; band structure; and crystallographic data (PDF)
