# Supplementary material for: Detailed Structural Features of the Perovskite-Related Halide RbPbI3 for Solar Cell Applications
Source: Inorg Chem. 2022 Mar 28;61(14):5502–11. doi: 10.1021/acs.inorgchem.1c03841 (PMC9006220; doi:10.1021/acs.inorgchem.1c03841)
Supplement: Supplementary file 1 — ic1c03841_si_001.pdf [file ic1c03841_si_001.pdf]

SUPPORTING INFORMATION

Detailed structural features of the perovskite-related halide $RbPbI_3$ for solar cell applications.

Carmen Abia,[a,b] Carlos A. López[a,c], Javier Gainza*[a], João Elias F. S. Rodrigues,[a,d] Mateus Ferrer[e], Gustavo Dalenogare[e], Norbert M. Nemes[a,f], Oscar J. Dura,[g] José L. Martínez[a], María T. Fernández-Díaz[b], Consuelo Álvarez-Galván[h], and José A. Alonso*[a]

[a.] *Instituto de Ciencia de Materiales de Madrid, CSIC, Cantoblanco 28049 Madrid, Spain.*
[b.] *Institut Laue Langevin, BP 156X, F-38042 Grenoble, France.*
[c.] *Instituto de Investigaciones en Tecnología Química (UNSL-CONICET) and Facultad de Química, Bioquímica y Farmacia, Almirante Brown 1455 (5700) San Luis, Argentina.*
[d.] *European Synchrotron Radiation Facility (ESRF), 71 Avenue des Martyrs, 38000 Grenoble, France.*
[e.] *CCAF, PPGCEM/CDTec, Federal University of Pelotas, 96010-610 Pelotas, Rio Grande do Sul, Brazil.*
[f.] *Departamento de Física de Materiales, Universidad Complutense de Madrid, E-28040 Madrid, Spain.*
[g.] *Departamento de Física Aplicada, Universidad de Castilla-La Mancha, Ciudad Real, E-13071, Spain.*
[h.] *Instituto de Catálisis y Petroleoquímica, CSIC, Cantoblanco 28049 Madrid, Spain.*
**ja.alonso@icmm.csic.es; j.gainza@csic.es.*

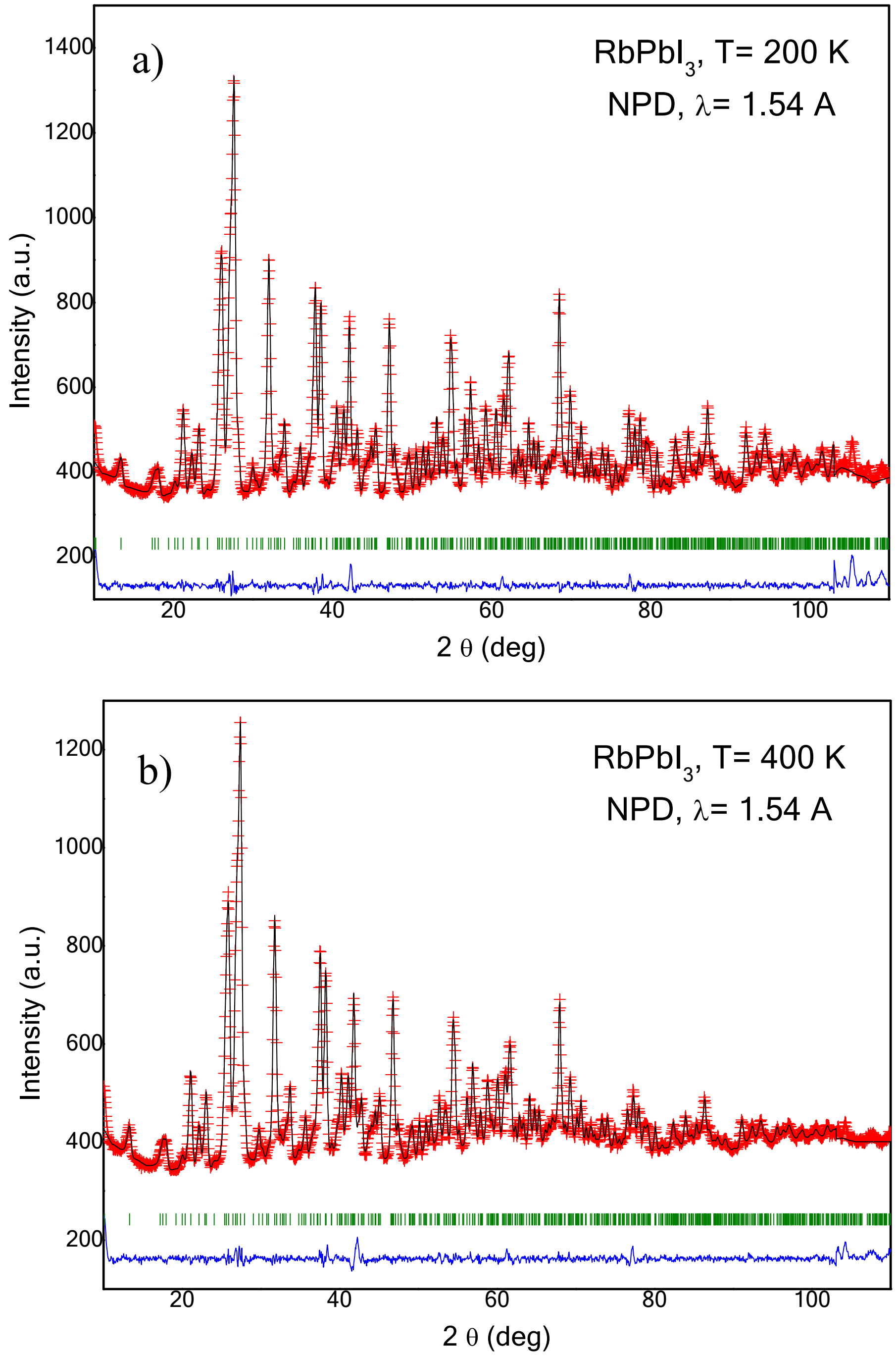

Figure S1. Observed (crosses) calculated (black line) and difference (blue line) profiles after the Rietveld refinement in the *Pnma* structure from NPD data, at 200 K (upper image) and 400 K (lower image).

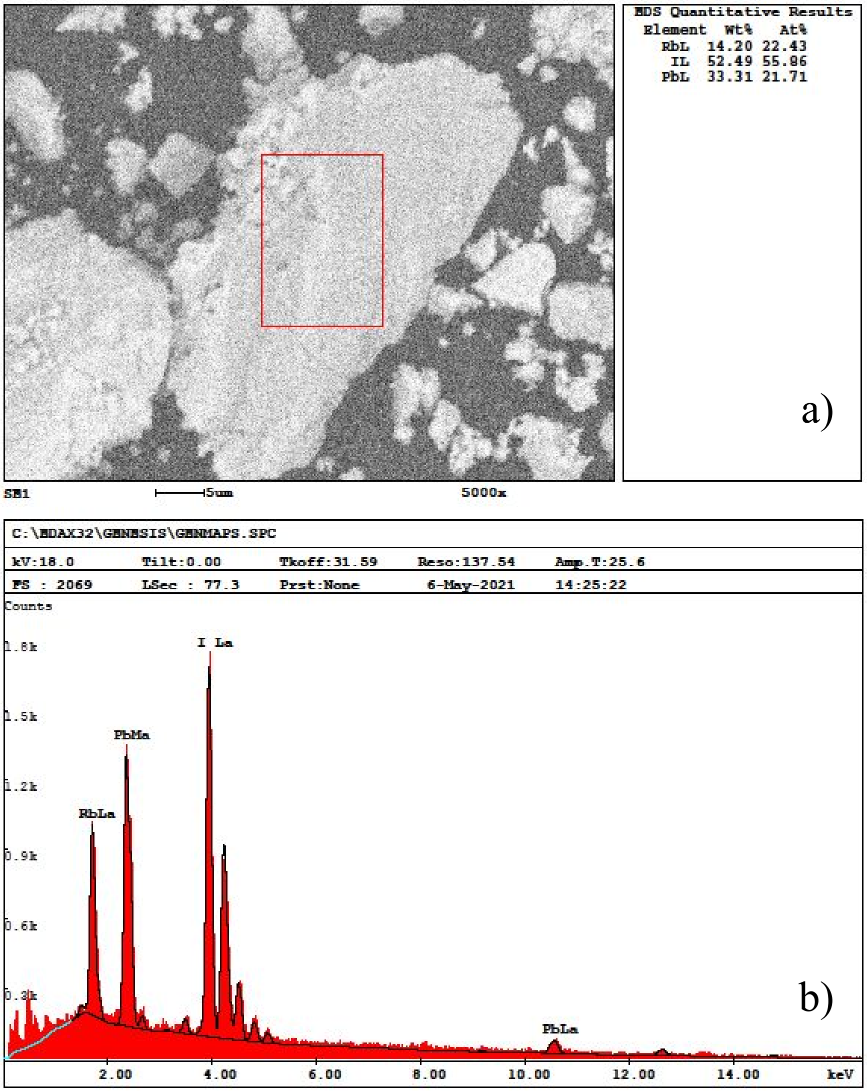

c)

Element	Determined	Stoich.
Rubidium	1.03	1.00
Lead	1.00	1.00
Iodine	2.57	3.00

Figure S2. a) Selected SEM image where EDX is collected, b) EDX spectrum with the atomic assignment, c) atomic ratio of the three elements (Rb, Pb, I).

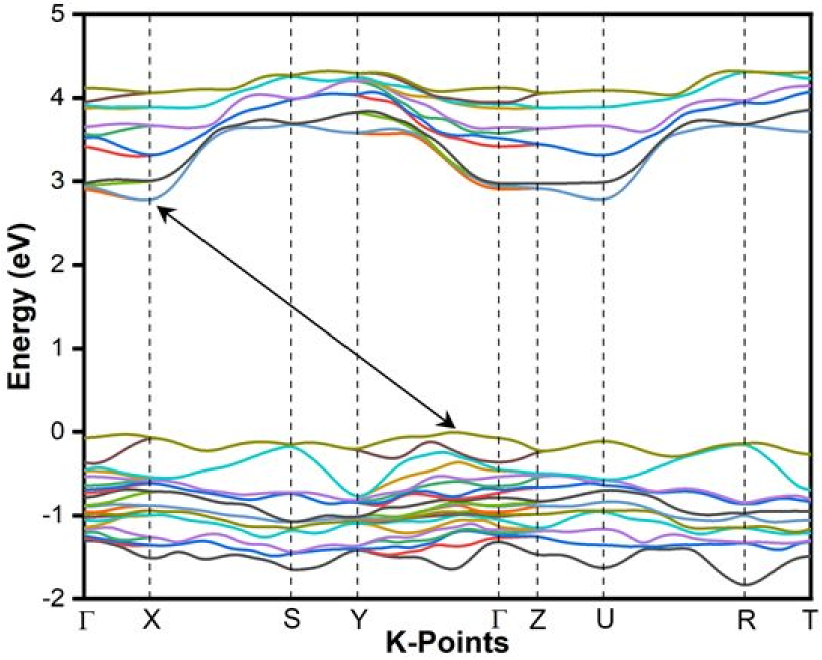

Figure S3. Band structure of $RbPbI_3$ model, showing an indirect band gap transition of 2.61 eV.

Table S1. Crystallographic data for $RbPbI_3$ phase in orthorhombic system (*Pnma*) from NPD data at 200 K.

Crystal data

Orthorhombic, *Pnma*	NPD, λ= 1.54 Å
a = 10.2195 (2) Å	b = 4.75055 (9) Å
c = 17.2932 (4) Å	V = 839.56 (3) Å^3
Z = 4	

Refinement

R_p = 0.92%	R_{wp} = 1.18%
R_{exp} = 0.73%	R_{Bragg} = 1.57%
χ^2 = 2.58	

Fractional atomic coordinates and isotropic or equivalent isotropic displacement parameters (Å^2)

	x	y	z	U_{eq} (Å^2)
Rb	0.4135 (2)	0.25000	0.67424 (16)	0.0331 (18)
Pb	0.16598 (17)	0.25000	0.43977 (10)	0.0237 (13)
I1	0.3059 (3)	0.25000	0.2846 (2)	0.028 (2)
I2	0.1586 (3)	0.25000	0.00898 (19)	0.025 (2)
I3	0.0277 (3)	0.25000	0.61765 (17)	0.022 (2)

Anisotropic displacement parameters (Å^2)

	U^{11}	U^{22}	U^{33}	U^{12}	U^{13}	U^{23}
Rb	0.0369 (18)	0.0254 (17)	0.037 (2)	0.00000	−0.0014 (15)	0.00000
Pb	0.0267 (12)	0.0241 (13)	0.0203 (12)	0.00000	0.0048 (13)	0.00000
I1	0.041 (2)	0.029 (2)	0.014 (3)	0.00000	0.000 (2)	0.00000
I2	0.019 (2)	0.017 (2)	0.040 (3)	0.00000	0.012 (2)	0.00000
I3	0.014 (3)	0.024 (2)	0.028 (2)	0.00000	−0.0054 (17)	0.00000

Table S2. Crystallographic data for $RbPbI_3$ phase in orthorhombic system (*Pnma*) from NPD data at 400 K.

Crystal data

Orthorhombic, Pnma	NPD, λ= 1.54 Å
a = 10.3007 (4) Å	b = 4.78626 (14) Å
c = 17.4186 (5) Å	V = 858.77 (5) $Å^3$
Z = 4	

Refinement

R_p = 0.87%	R_{wp} = 1.19%
R_{exp} = 0.72%	R_{Bragg} = 2.07%
χ^2 = 2.71	

Fractional atomic coordinates and isotropic or equivalent isotropic displacement parameters ($Å^2$)

	x	*y*	*z*	U_{eq} ($Å^2$)
Rb	0.4138 (4)	0.25000	0.6734 (2)	0.073 (3)
Pb	0.1652 (3)	0.25000	0.43907 (15)	0.048 (2)
I1	0.3071 (5)	0.25000	0.2845 (3)	0.057 (4)
I2	0.1607 (6)	0.25000	0.0100 (3)	0.061 (4)
I3	0.0270 (4)	0.25000	0.6157 (2)	0.039 (4)

Atomic displacement parameters ($Å^2$)

	U^{11}	U^{22}	U^{33}	U^{12}	U^{13}	U^{23}
Rb	0.087 (3)	0.064 (3)	0.068 (3)	0.00000	−0.001 (2)	0.00000
Pb	0.061 (2)	0.041 (2)	0.0407 (19)	0.00000	0.001 (2)	0.00000
I1	0.075 (4)	0.055 (4)	0.041 (4)	0.00000	0.019 (3)	0.00000
I2	0.074 (5)	0.053 (4)	0.057 (4)	0.00000	0.030 (3)	0.00000
I3	0.037 (4)	0.038 (3)	0.043 (4)	0.00000	−0.001 (3)	0.00000
